# Optimal dose of lactoferrin reduces the resilience of in vitro *Staphylococcus aureus* colonies

**DOI:** 10.1371/journal.pone.0273088

**Published:** 2022-08-12

**Authors:** Jagir R. Hussan, Stuart G. Irwin, Brya Mathews, Simon Swift, Dustin L. Williams, Jillian Cornish

**Affiliations:** 1 Auckland Bioengineering Institute, University of Auckland, Auckland, NZ; 2 Department of Molecular Medicine and Pathology, University of Auckland, Auckland, NZ; 3 Department of Microbiology and Immunology, School of Medicine, University of Utah, Salt Lake City, Utah, United States of America; 4 Department of Medicine, University of Auckland, Auckland, NZ; Bhabha Atomic Research Centre, INDIA

## Abstract

The rise in antibiotic resistance has stimulated research into adjuvants that can improve the efficacy of broad-spectrum antibiotics. Lactoferrin is a candidate adjuvant; it is a multifunctional iron-binding protein with antimicrobial properties. It is known to show dose-dependent antimicrobial activity against *Staphylococcus aureus* through iron sequestration and repression of *β*–lactamase expression. However, *S. aureus* can extract iron from lactoferrin through siderophores for their growth, which confounds the resolution of lactoferrin’s method of action. We measured the minimum inhibitory concentration (MIC) for a range of lactoferrin/ *β*–lactam antibiotic dose combinations and observed that at low doses (< 0.39 *μM*), lactoferrin contributes to increased *S. aureus* growth, but at higher doses (> 6.25 *μM*), iron-depleted native lactoferrin reduced bacterial growth and reduced the MIC of the *β*-lactam-antibiotic cefazolin. This differential behaviour points to a bacterial population response to the lactoferrin/ *β*–lactam dose combination. Here, with the aid of a mathematical model, we show that lactoferrin stratifies the bacterial population, and the resulting population heterogeneity is at the basis of the dose dependent response seen. Further, lactoferrin disables a sub-population from *β*-lactam-induced production of *β*-lactamase, which when sufficiently large reduces the population’s ability to recover after being treated by an antibiotic. Our analysis shows that an optimal dose of lactoferrin acts as a suitable adjuvant to eliminate *S. aureus* colonies using *β*-lactams, but sub-inhibitory doses of lactoferrin reduces the efficacy of *β*-lactams.

## Introduction

*Staphylococcus aureus* is the leading cause of soft tissue, device-related and surgical wound infections [1]. It adheres to tissue and abiotic surfaces, like prosthetics, and develops metabolically frugal biofilms that are tolerant of antibiotics [2, 3]. Subpopulations of *S. aureus* cells are known to exhibit a phenotype with reduced growth rate and altered gene expression; these include persisters, and small colony variants (SCV) [4]. Biofilm formation is a key part of establishing chronic infection [5, 6], with an increased tolerance to antibiotic therapies [7]. Achieving antibiotic concentrations capable of persister cell eradication in vivo can be difficult or impossible [8]. Increasing the dose and the use of antibiotics also poses the risk of developing of drug resistant strains. For instance, *β*-lactam antibiotics are very effective against fast growing bacterial cells but are ineffective against bacterial cells that are not actively dividing and synthesizing new cell wall peptidoglycans [9].

*β*-lactam antibiotics have been a mainstay of clinical therapeutics, especially for methicillin-susceptible *S. aureus* (MSSA) infections as they are highly effective against non-resistant pathogens, have a lower risk of side effects, and are less expensive, relative to second-line antibiotics [10]. However, the concern that sustained *β*-lactam use could promote horizontal gene transfer of virulence factors to other microorganisms [11] has increased attention on developing alternative antibiotic treatments.

Bacteria develop tolerance through two primary mechanisms: 1) through adaptive stress responses that reduce the efficacy of the antibiotic (such as prevent binding, or actively degrading the antibiotic) [12, 13]; 2) through stratification of the population into tolerance phenotypes with some subpopulations that are highly tolerant of the antibiotic and transition between these phenotypes occur based on the level of stress [14, 15]. *S. aureus* strains are known to produce *β*-lactamase enzymes to degrade *β*-lactams.

Therefore, we hypothesise that complementing *β*-lactam antibiotic with an adjuvant that reduces the impact of the adaptive response (*β*-lactamase production) will result in improved efficacy of the antibiotic and consequently require lower *β*-lactam dose. In this context, we explored whether lactoferrin, a naturally occurring iron-binding protein with many biological functions, including bacteriostatic, and immunomodulatory activities could be used as an adjuvant. Lactoferrin has multiple activities that contribute to antimicrobial activity. Firstly, it can sequester iron that is essential for bacterial growth. Secondly, lactoferrin and its peptide derivatives bind to bacterial components and can disrupt bacterial function and membrane integrity [16]. Studies on *Pseudomonas aeruginosa* have shown that lactoferrin disrupts biofilm formation through a combination of effects that degrade the biofilm matrix and stimulate dispersal; these include chelating iron, stimulating twitching motility, interfering with cell-to-cell signalling processes, increasing DNase activity, and reducing bacterial glycosidase activity [17, 18].

Investigating the combined effect of lactoferrin and *β*-lactam is not straightforward as MSSA strains can overcome inhibitory and lethal actions of lactoferrin and degrade *β*-lactam. *S. aureus* cultures typically consist of subpopulations due to heterogeneous availability of nutrients, which necessitates altered metabolic phenotypes. Of particular interest are slow growing or non-growing cells, stationary-phase cells and persisters [9, 19]. In this paper, such slow growing and/or non-growing cells are collectively referred to as *Persister cells*. Persister cells that exist within a population enable recovery given time; an essential dynamic that needs to be characterised to quantify efficacy of a treatment. In the presence of persisters, bacterial growth dynamics such as the rate of growth and time to recovery provide a more realistic insight into the efficacy of the treatment. The complexity associated with experimentally tracking these subpopulations and measuring the time course data for all the lactoferrin and *β*-lactam treatment combinations required us to explore alternative methods to infer this information. Here we used a computational model to infer this information.

There are several mathematical models in published literature that incorporate the dynamics of susceptible and persister populations [20–24]. These models focus on the role played by adaptive responses that prevent antibiotic binding, persister formation and reversion, and the time scale of antibiotic treatments. We did not find any mathematical models in published literature that investigated the role of lactoferrin or any adjuvant on the efficacy of the antibiotic. Since our goal is to determine the role played by lactoferrin in altering the adaptive response of bacteria, specifically does lactoferrin reduce *β*-lactamase production; if it does then determine the extent to which *β*-lactamase production must be reduced for a *β*-lactam dose to be effective. To achieve this, we extended a published model on *β*-lactam antibiotic activity [24] to predict the growth dynamics from the experimental data, and estimate the role of lactoferrin in the *β*-lactamase production dynamics.

Here, we show the response of *S. aureus* broth cultures to various combinations of *β*-lactam antibiotic (Cefazolin) and lactoferrin doses at different iron saturation levels, model predictions of temporal dynamics of the bacterial population in response to these doses, and the potential synergistic role of lactoferrin in reducing the tolerance of the population.

## Materials and methods

### Materials preparation

*Staphylococcus aureus* Xen36 (PerkinElmer, Part Number 119243), a methicillin sensitive, beta-lactamase positive strain [25], that is engineered for bioluminescence to facilitate in vivo infection studies is used as a representative isolate [26]. Antibiotic assays were performed in BBL Cation-Adjusted Mueller Hinton II Broth (MHB; Fort Richard, Auckland). Cefazolin was purchased from Sigma-Aldrich. Native bovine lactoferrin was supplied by Fonterra, NZ. Iron-loaded lactoferrin (approximately 80% iron saturation) was prepared by incubating 100 mg/ml native lactoferrin with 2× molar equivalents of Fe_2_Cl_3_ and NaHCO_3_ for 24 hours as described in [27], followed by three 12-hour rounds of dialysis against 40 volumes of PBS to remove unbound iron and return to a neutral pH. Protein content was calculated from HPLC data (Fonterra) and Fe^3+^ content was verified using inductively coupled plasma-mass spectrometry (Agilent 7700 ICP-MS in He mode).

### Bacterial cultures

Bacterial cultures were set by adding two to three colonies of *S. aureus* Xen36 to 10 ml of MHB in a 50 ml conical tube and incubated overnight at 37°C with shaking at 200 RPM. Broths were assessed by absorbance at 600 nm, comparing doubling dilutions against a previously established standard curve, and diluted to the required cell density.

### Checkerboard assay

A checkerboard assay was used to measure synergistic/inhibitory interactions of lactoferrin preparations with the antibiotic cefazolin against *S. aureus* Xen36. A two-dimensional, two-agent (cefazolin and lactoferrin), doubling microdilution checkerboard was prepared in a 96-well microplate [28] in sterile MilliQ water, with each well containing 50 *μl* of the reagent combinations. An overnight culture of *S. aureus* Xen36 grown in MHB was diluted to 2 × 10^5^ CFU/ml in 2× MHB, and 50 *μl* was added to each well of the microplate to give a final volume of 100 *μl* in 1 × MHB (containing approximately 14 *μ*M Fe) to challenge an inoculum of 1 × 10^5^ CFU/ml with the various cefazolin/lactoferrin combinations. Cefazolin was tested in a range from 0 to 4.0 *μ*g/ml. Lactoferrin preparations (native, iron-loaded, and a 1:1 mixture of native:iron-loaded) were tested in a range from 0 to 100 *μ*M. Optical measurements of absorbance at 600 nm and of bioluminescence (EnSpire^®^ Multimode Plate Reader, PerkinElmer, USA) were taken prior to incubation and after 16 ± 2 h incubation at 37°C with humidity and shaking at 200 RPM. Each checkerboard assay plate was replicated on three separate occasions, data is available in S1 Data.

Absorbance values were used to calculate minimum inhibitory concentrations MIC50 (where growth is inhibited by ≥ 50% of the no antibiotic control) and MIC90 (where growth is inhibited by ≥ 90% of the no antibiotic control) for antibiotic alone and antibiotic in the presence of lactoferrin preparations. Estimations of MIC50 and MIC90 were also made using bioluminescence measurements.

### Kinetic model development

**Fig 1:**
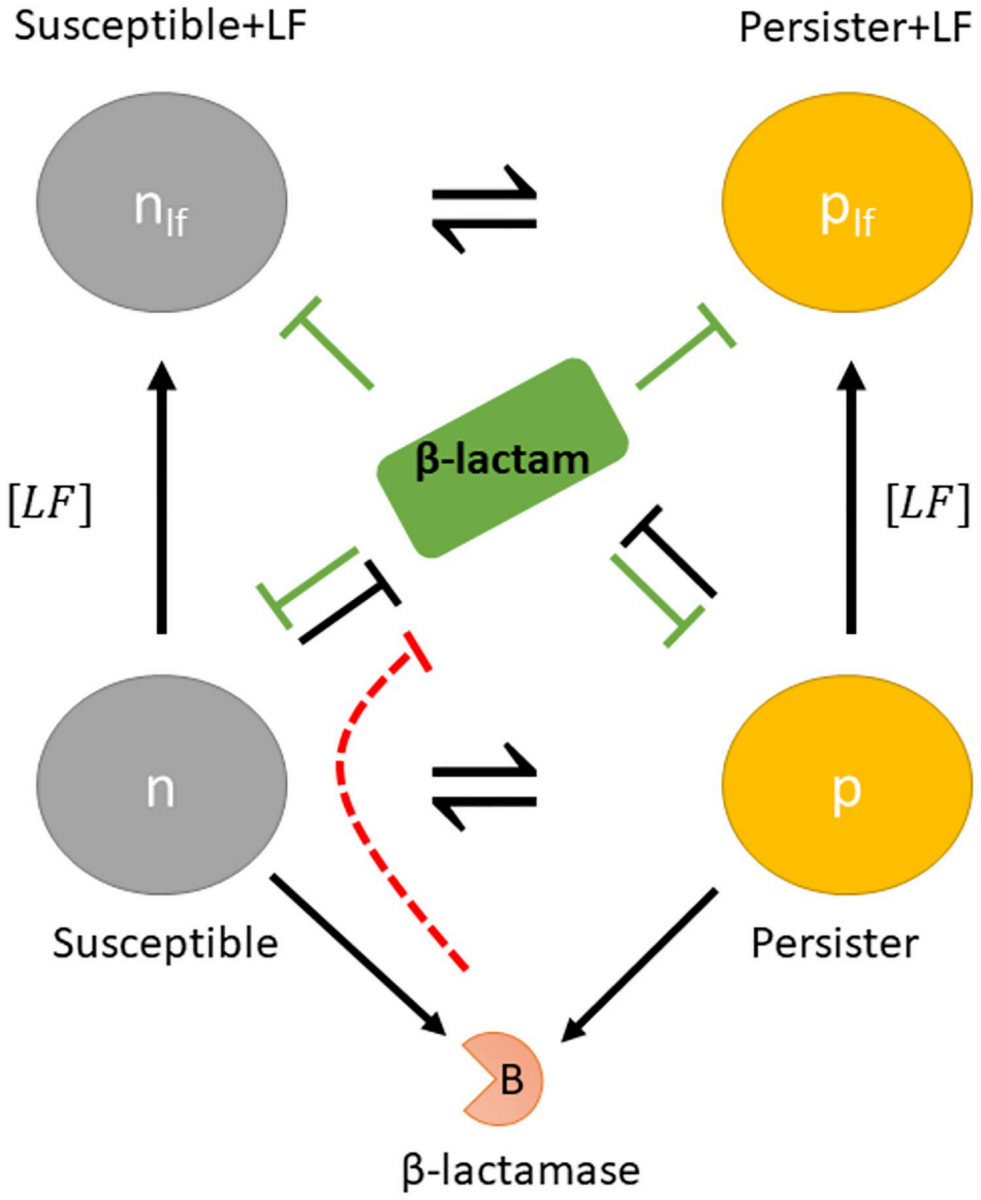
Kinetic model of lactoferrin/ *β*-lactam interaction with bacterial population that produces *β*-lactamase when treated with an antibiotic.

We developed a mathematical model to examine population based response to lactoferrin/ *β*-lactam antibiotic treatment. The model is based on the observations that, on exposure to *β*-lactam antibiotic, *β*-lactamase producing bacteria like *S. aureus* express *β*-lactamase and degrade the bound antibiotic which reduces the antibiotic’s efficacy. Further, bacterial lysis releases extra-cellular *β*-lactamase into the surroundings, which further confers protection to the population by degrading the antibiotic in the surroundings. Our model focuses on a mixed heterogeneous population of slow and fast growing cells that constitutively expresses *β*-lactamase when no lactoferrin is bound to the cells. The model accounts for growth rates of the subpopulations, stress induced partitioning of population and *β*-lactamase production in response to antibiotic and lactoferrin induced stress. Modelling the population density response provides a level of abstraction that captures the contributions of multiple cell level interactions. A schematic of the interactions characterised by the model is shown in Fig 1.

The model characterises the growth and lysis dynamics of four types of bacterial subpopulations: susceptible (*n*, *n*_*lf*_) and persister (*p*, *p*_*lf*_) cells. Lactoferrin free sub-population (*n*, *p*) can produce *β*-lactamase in the presence of *β*-lactam. The remaining population (*n*_*lf*_, *p*_*lf*_) to which lactoferrin is bound, is repressed from expressing *β*-lactamase. The density of the repressed population depends on the concentration of extracellular lactoferrin. The model accounts for the variability in growth rates (*g*, *g*_*p*_), lysis rates (*l*, *lf*_*l*_, *l*_*p*_, *lf*_*lp*_) and *β*-lactamase expression between subpopulations. Note that the growth rates of both lactoferrin bound and unbound susceptible and persister cells share the same growth rates respectively, however have the lysis rates are different to characterise the complex antibiotic and *β*-lactamase interactions for each cell-type.

We adopted and extended previously published models [22, 24] to account for the dynamics of bacterial densities, extracellular *β*-lactamase concentration (*b*_*out*_), membrane bound *β*-lactamase concentration (*b*_*in*_), lactoferrin concentration (*Lf*), and *β*-lactam concentration (*A*). To model the capability of *S. aureus* to extract iron from lactofferin and support its growth/maintenance, the modelled growth rates depend on lactoferrin’s iron saturation level. The amount of membrane bound *β*-lactamase concentration (*b*_*in*_) produced by the bacterial cells is proportional to the available *β*-lactam concentration. The amount of extracellular *β*-lactamase concentration (*b*_*out*_) is determined by the lysis rate of the *β*-lactamase producing cells. The model makes the following assumptions

1. The cell density for each subpopulation (*n*, *n*_*lf*_, *p*, *p*_*lf*_) depends on its growth rate (*g*, *g*_*p*_) and their corresponding lysis rate (*l*, *lf*_*l*_, *l*_*p*_, *lf*_*lp*_),
2. The entire initial population can produce *β*-lactamase in the presence of *β*-lactam,
3. The growth rates are a function of the maximum growth rate of the subpopulation (*σ*_1_, *σ*_5_),
4. There is sufficient nutrient available to the population at all times. The nutrient level was set constant,
5. The population level *β*-lactamase concentration (*b*_in*_) was determined by multiplying the number of *β*-lactamase producing cells present at a given time and per cell *β*-lactamase concentration (*b*_*in*_) and the averaged volume of a bacterial cell, *β*,
6. Lactoferrin bound to the bacteria are not recovered when the bacteria to which they are bound lyse,
7. The lysis rate of bacteria to which lactoferrin is bound is a function of the antibiotic concentration and lysis rates (*σ*_2_, *σ*_6_), and
8. The lysis rate of bacteria free of lactoferrin is a function of the antibiotic concentration, lysis rates (*σ*_2_, *σ*_6_), and amount of membrane bound *β*-lactamase *b*_*in*_.

The non-dimensionalised model equations are

$$\begin{array}{cc} \frac{dn}{dt} & =g\cdot \left(n+n_{lf}\right)-l\cdot n+\kappa_{N}\cdot p-\kappa_{P}\cdot n-lf\cdot n-\delta_{\beta }\cdot n\\ \frac{dp}{dt} & =g_{p}\cdot \left(p+p_{lf}\right)-l_{p}\cdot p-\kappa_{N}\cdot p+\kappa_{P}\cdot n-lf\cdot p-\delta_{p}\cdot p\\ \frac{dn_{lf}}{dt} & =-lf_{l}\cdot n_{lf}+\kappa_{N}\cdot p_{lf}-\kappa_{P}\cdot n_{lf}+lf\cdot n-\delta_{\beta }\cdot n_{lf}\\ \frac{dp_{lf}}{dt} & =-lf_{l}p\cdot p_{lf}-\kappa_{N}\cdot p_{lf}+\kappa_{P}\cdot n_{lf}+lf\cdot p-\delta_{p}\cdot p_{lf}\\ \frac{db_{out}}{dt} & =b_{in}^{*}\cdot \left(l+l_{p}\right)-\gamma_{2}\cdot b_{out}\\ \frac{dA}{dt} & =-\gamma_{6}\cdot \left(b_{out}+\alpha \cdot b_{in}^{*}\right)\frac{A}{\left(1.0+A\right)}-\gamma_{3}\cdot A\\ \frac{dLf}{dt} & =-lf\cdot \left(n+p\right)-\gamma_{8}\cdot Lf\\ g & =lgf\cdot \left(1.0-n_{T}\right)\frac{\sigma_{1}}{\left(\sigma_{1}+A\right)}\frac{s}{\left(1.0+s\right)}\\ g_{p} & =lgf\cdot \left(1.0-n_{T}\right)\frac{\sigma_{5}}{\left(\sigma_{5}+A\right)}\frac{s}{\left(1.0+s\right)}\varphi \\ l & =\gamma_{1}\frac{A^{H}}{\left(\sigma_{2}^{H}+A^{H}\right)}\frac{\sigma_{4}}{\left(\sigma_{4}+b_{in}\right)}\\ l_{p} & =\gamma_{5}\frac{A^{H}}{\left(\sigma_{6}^{H}+A^{H}\right)}\frac{\sigma_{4}}{\left(\sigma_{4}+b_{in}\right)}\\ lf_{l} & =\gamma_{1}\frac{A^{H}}{\left(\sigma_{2}^{H}+A^{H}\right)}\\ lf_{lp} & =\gamma_{5}\frac{A^{H}}{\left(\sigma_{6}^{H}+A^{H}\right)}\\ lf & =\gamma_{7}\frac{Lf^{H}}{\left(\sigma_{7}^{H}+Lf^{H}\right)}\\ lgf & =1.0+H\left(Lf\right)\left[Fe^{3+}\right]\frac{\sigma_{8}^{H}}{\left(\sigma_{8}^{H}+Lf^{H}\right)}\\ n_{T} & =n+p+n_{lf}+p_{lf}\\ r & =\frac{A}{\left(\sigma_{3}+A\right)}\\ b_{in} & =\kappa \cdot (r\cdot \left(g+g_{p}+\gamma_{4}\right))\\ b_{in}^{*} & =\beta \cdot b_{in}\cdot \left(n+p\right) \end{array}$$

Here H(*Lf*) is the Heaviside function. The physical interpretation of the parameters are given in Table 1.

**Table 1:** Non dimensionalised model parameters.

Name	Value	Description
*β*	0.0034	Scale factor for converting single cell *β*-lactamase production to that of the population.
*γ*_1_	75.0551	Maximum lysis rate of susceptible bacteria by antibiotic.
*γ*_2_	1.0686	Maximum degradation rate of extracellular *β*-lactamase.
*γ*_3_	0.3564	Maximum degradation rate of *β*-lactam antibiotic.
*γ*_4_	0.4135	Maximum degradation rate of membrane bound *β*-lactamase.
*γ*_5_	0.0559	Maximum lysis rate of persister bacteria by antibiotic.
*γ*_6_	0.5524	Maximum degradation rate of antibiotic by *β*-lactamase.
*γ*_7_	0.7868	Maximum rate of free bacterial attachment of lactoferrin.
*γ*_8_	0.5362	Maximum degradation rate of extracellular lactoferrin.
*ν*	0.0031	Growth rate of persister population with respect to susceptible cells.
*κ*_*N*_	1.0354	Maximum rate at which persister cells switch to susceptible cells.
*κ*_*P*_	0.7809	Maximum rate at which susceptible cells switch to persister cells.
*σ*_1_	0.2434	Half maximal constant for growth inhibition by antibiotic.
*σ*_2_	1.8814	Half maximal constant for lysis of susceptible cells by antibiotic.
*σ*_3_	4.2311	Half maximal constant for inducing *β*-lactamase production.
*σ*_4_	2624.2065	Half maximal constant for membrane bound *β*-lactamase protection from antibiotic.
*σ*_5_	4.0515	Half maximal constant for persister cell growth inhibition.
*σ*_6_	0.3912	Half maximal constant for lysis of persister cells by antibiotic.
*σ*_7_	2.5834	Half maximal constant for lactoferrin binding to free bacteria.
*σ*_8_	0.4229	Half maximal constant for lactoferrin induced growth inhibition.
H	4.0	Hill Coefficient (to capture time dependence).
*δ*_*β*_	1.9914	Natural death rate of susceptible cells.
*δ*_*p*_	0.5997	Natural death rate of persister cells.
Kv	0.5139	Volumetric non dimensionalisation coefficient. Injected antibiotic and lactoferrin concentrations are divided by Kv prior to adding it to the state values to simulate dose administration.
A	0.0087	Weighting factor for membrane bound *β*-lactamase, which provides less protection against antibiotic than extracellular *β*-lactamase.
K	3312.3079	Efficiency of *β*-lactamase to hydrolyse antibiotic.
s	7.1800	Nutrient concentration.
[Fe^3+^]–Native	1.3932	Maximum Fe^3+^ saturation induced growth for Native lactoferrin.
[Fe^3+^]–Mixed	1.4441	Maximum Fe^3+^ saturation induced growth for 50% Native + 50% Fe^3+^ saturated lactoferrin.
[Fe^3+^]–Saturated	1.4554	Maximum Fe^3+^ saturation induced growth for Fe^3+^ saturated lactoferrin.

#### Parameter fitting

**Fig 2:**
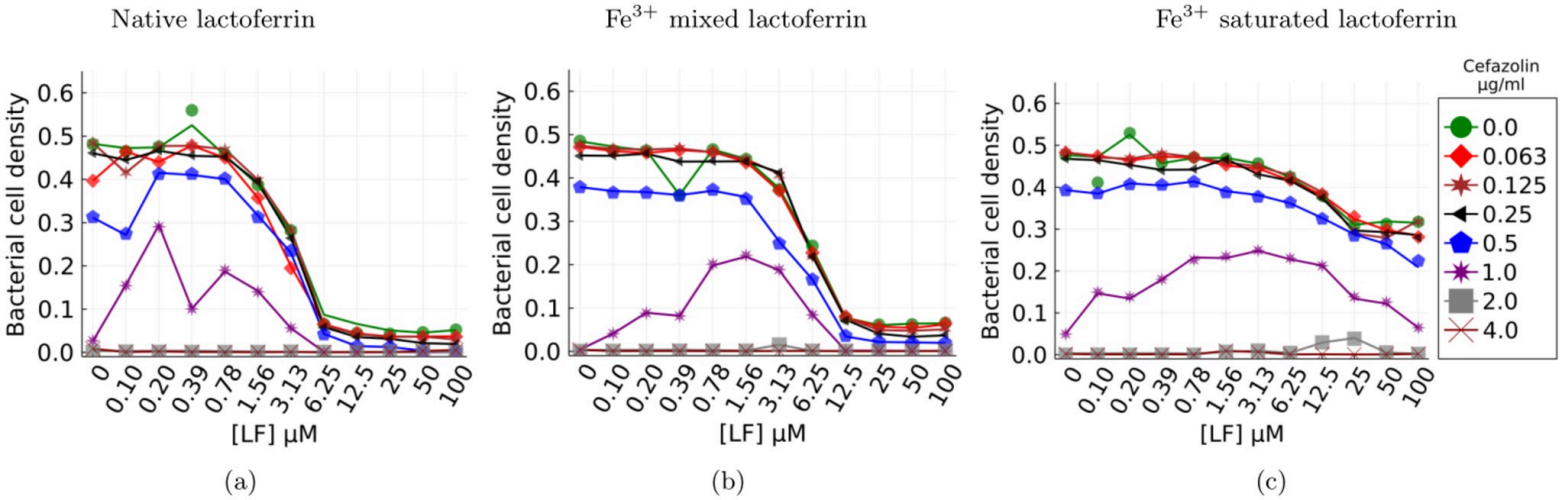
Kinetic model prediction vs experimental data. The kinetic ode model was parameterised to fit the experimental data, the model results (solid lines) are plotted against the experimental data (points). One model ([*Fe*^3+^]) parameter was a function of the lactoferrin Fe^3+^ saturation level, all others remained the same.

The model parameters were estimated using constrained optimization using linear approximations (COBYLA). The set of parameters that predict the growth values for all data sets was selected. Among the parameters, the parameter [Fe^3+^] that captures the growth induced by the lactoferrin due to its iron saturation level was matched to the data set. Parameters were further constrained to ensure that the solutions always remained in the positive numerical domain as there are no-negative densities or concentrations. For parameter estimation, the combined lactoferrin/ *β*-lactam dose was introduced when the total bacterial population reached steady state. The bacterial population kinetics from the resulting crash and recovery was used to fit the experimental predictions. The system ordinary differential equations are stiff and were solved using Rosenbrock23 ODE solver. All simulations were performed using Julia v 1.6, DifferentialEquations package v 6.19 and NLopt v 0.6.2. The model parameters are listed in Table 1 and the results are plotted in Fig 2.

#### Parameter sensitivity analysis

The proposed system of equations have 28 non-dimensional parameters. While the low RMSD (root mean square deviation) of fit (Fig 2) provides some confidence; however, in models with large number of parameters the potential to find more than one dissimilar set of parameters that generate similar model outputs is highly likely [29]. Sensitivity analysis allows the identification of the set of parameters that have the greatest influence on the model output. It consequently provides useful insight into which model parameters contribute most to the variability of the model output, and identifying the important and influential parameters that drive model outputs and magnitudes. Here, in addition to the low RMSD, we constrained the parameter search to find a parameter set where key parameters that affect the bacterial population as a function of Cefazolin and lactoferrin had the greatest influence. Following [30] we used sobol sensitivity analysis to determine the total-order sensitivity index, 0 ≤ *ST* ≤ 1.0. *ST* characterises a parameter’s contribution to the variation in model’s predictions, alone or through the interaction with any number of other parameters. Sobol sensitivity analysis was performed on the dimensionless model, on a parameter space centred around each parameter’s fitted value(*x*_0_), spanning $\left(x\in \left[\frac{x_{0}}{2},2x_{0}\right]\right)$. Using a quasi-montecarlo approach a design matrix of 5000 samples of the parameter space were evaluated using Julia v 1.6, GlobalSensitivity package v 1.3. The parameters with the leading total-order sensitivity index values are listed in Table 2.

**Table 2:** Modal parameters with leading total-order sensitivity index values.

Parameter	Total-order Index	Description
*δ*_*p*_	0.9774	Natural death rate of persister cells.
*δ*_*β*_	0.9489	Natural death rate of susceptible cells.
[Fe^3+^]-	0.9141	Maximum Fe^3+^ saturation induced growth
*κ*_*P*_	0.8496	Maximum rate at which susceptible cells switch to persister cells.
*lf*	0.8310	Lactoferrin concentration dependent rate of switching from lactoferrin-free to lactoferrin-bound bacteria.
*κ*_*N*_	0.6841	Maximum rate at which persister cells switch to susceptible cells.
*ν*	0.5245	Growth rate of persister population with respect to susceptible cells.
*σ*_1_	0.4859	Half maximal constant for growth inhibition by antibiotic.
*γ*_8_	0.4839	Maximum degradation rate of extracellular lactoferrin.

These results in Table 2 show that parameters associated with population death rates (*δ*_*p*_, *δ*_*β*_), Lactoferrin’s iron saturation induced growth [Fe^3+^]−, rates of bacterial population switching (*κ*_*N*_, *κ*_*P*_), lactoferrin binding (*lf*), persister growth rate fraction (*ν*), and degradation rate of extracellular lactoferrin (*γ*_8_) have the highest index values. Note that *lf*, depends on the parameters *γ*_7_, *H* and *σ*_7_. Since $ST_{i}=\frac{V_{i}}{Var(Y)}$, where *V*_*i*_ is variance due to parameter *i* alone and *Var*(*Y*) is the variance due to the all parameter interactions. *ST*_*lf*_ was calculated as $3\times (ST_{\gamma_{7}}+ST_{H}+ST_{\sigma_{7}})$, using the variance sum law. The scaling factor of 3 is to account for the triple counting of *Var*(*Y*) in the denominator of the sum.

The results suggest that the fitted set of parameters enforce the proposed behaviour (Fig 1), suggesting that the observed response arises from the bacterial population behaviour.

#### Experimental validation

We used the model to predict the bacterial densities at 24 h post treatment for a range of Cefazolin and native lactoferrin doses to identify minimum bactericidal concentration (MBC). The model predicted a small range of doses around Cefazolin 0.75 *μ*g/ml and native lactoferrin dose of 18.0 *μ*M to be optimal. We selected 0.75 *μ*g/ml and 18.0 *μ*M as it was not used in the fitting of model parameters.

To validate this prediction, Xen36 bacteria grown in MHB was diluted to 2×10^5^ CFU/ml in 2× MHB, and 50 *μl* (identical to the preparation used in the Checkerboard assay experiments) were challenged with

1. combined doses of Cefazolin 0.5, 0.75, 1.0 *μ*g/ml, and Native lactoferrin 18.0 *μ*M,
2. just Cefazolin 0.5, 0.75, 1.0 *μ*g/ml, and
3. just Native lactoferrin 18.0 *μ*M

in triplicate. A control sample without any treatment was also setup.

After 24 h incubation, 10.0 *μ*l of the broth was plated on TSA agar plate (one plate per well), incubated for 24 h and CFU’s were counted. The experimental results, Table 3, showed that the combined dose of (Cefazolin 0.75 *μ*g/ml, and Native lactoferrin 18.0 *μ*M) eliminated the bacteria (CFU = 0 in all replicates), while separate doses recovered viable CFUs. The higher Cefazolin dose also eliminated the bacteria. These results further add confidence to the model and fit parameters.

**Table 3:** CFU counts from experimental treatment of Xen36 bacteria. Cells with • indicate too many CFU’s to count.

Cefazolin *μ*g/ml	0	0	0.5	0.75	1.0	0.5	0.75	1.0
Lactoferrin *μ*M	0	18.0	0	0	0	18.0	18.0	18.0
Rep 1	•	•	•	•	•	0	0	0
Rep 2	•	•	•	•	•	0	0	0
Rep 3	•	•	•	•	•	•	0	0

## Results

### *S. aureus* colony response to Cefazolin and lactoferrin treatment

**Fig 3:**
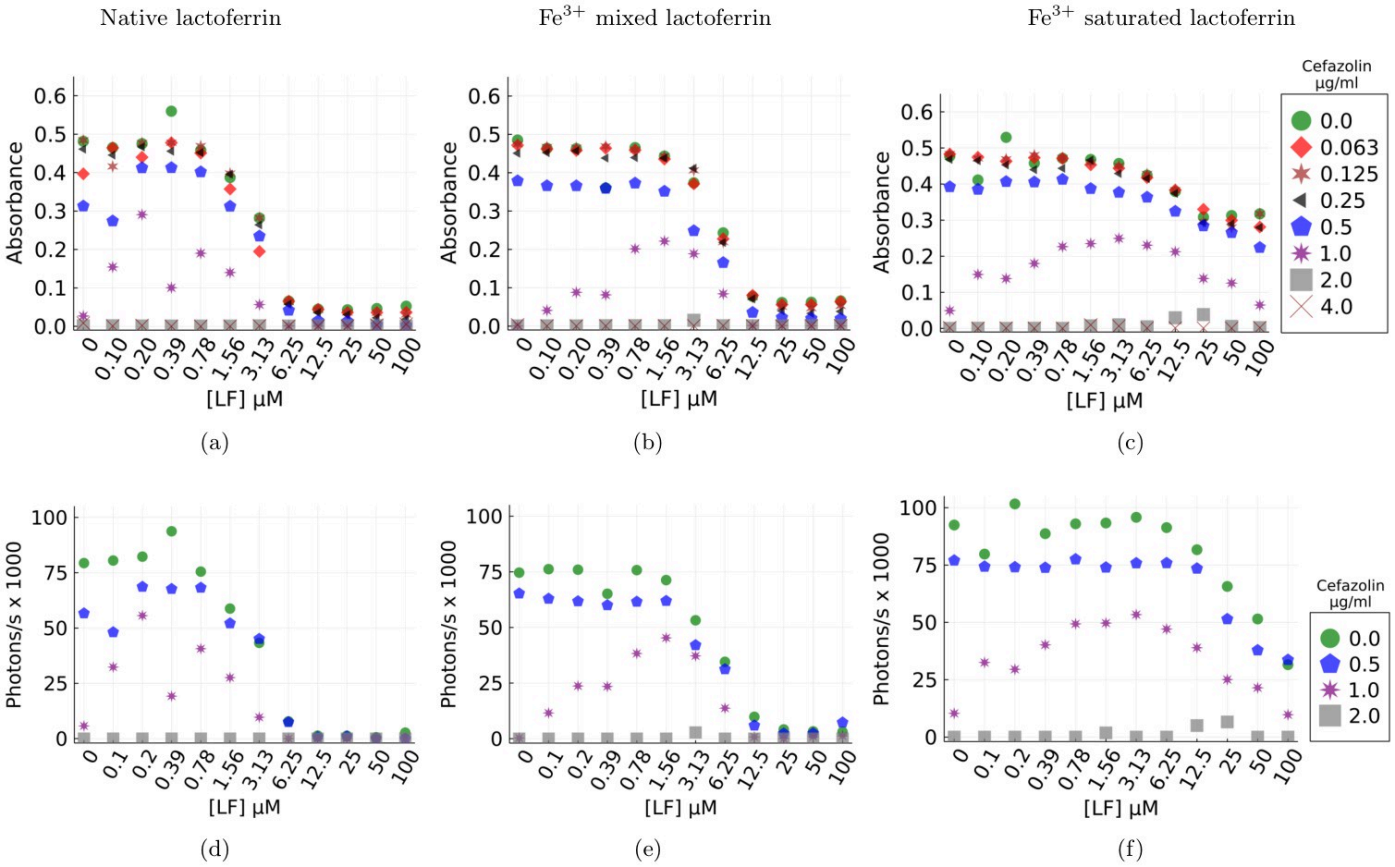
Absorbance and bioluminescence estimates of *S. aureus* 12–18 hours post treatment by Cefazolin+lactoferrin adjuvant. Markers correspond to concentrations of Cefazolin (eight for absorbance and four for bioluminescence). Each point on the graph represents a biological replicate of three estimates for that lactoferrin concentration. Bioluminescence qualitatively replicates the optical density-based estimate of the population for all the four combined treatments. The data highlights the nonlinear dependence of bacterial growth on lactoferrin adjuvance and its iron-saturation. a, d) Native lactoferrin b, e) Mixed lactoferrin c, f) Saturated lactoferrin.

Minimum inhibitory concentration (MIC) assays were performed with *S. aureus* Xen36. This strain can form biofilms, expresses *β*-lactamase and is bioluminescent. To investigate whether the inclusion of lactoferrin as an adjuvant would improve the antimicrobial activity of Cefazolin, a *β*-lactam antibiotic commonly used in orthopaedics, against *S. aureus* populations. We measured bacterial growth, after overnight incubation with different combinations of cefazolin and lactoferrin. To investigate the role of lactoferrin in terms of iron restriction, we compared native (15–19% iron saturation) lactoferrin, iron-saturated (80% iron saturation) lactoferrin and a 1:1 mixture of native and iron-saturated lactoferrin. The results show the baseline MIC50 and MIC90 for cefazolin alone were 1.0 and 2.0 *μ*g/ml respectively, and that native and mixed-native bacteriostatic were antimicrobial at MICs, but iron saturated lactoferrin was not obviously bacteriostatic in the test conditions (Fig 3). The MIC for cefazolin reduced to 0.5 *μ*g/ml in the presence of 6.25 *μ*M lactoferrin (Fig 3a and 3b).

Notably, there is enhanced bacterial survival when cefazolin concentrations of 1 *μ*g/ml (just below MIC) are combined with 0.1 − 1.5 *μ*M lactoferrin(Fig 3). We argue that the observed increase in growth is a consequence of the existence of both susceptible and persister cells. Sub-inhibitory concentrations of antibiotic elicits a stress response from the bacterial population, and some of the population switch to persister phenotype [31] thereby reducing the bactericidal activity of the *β*-lactam antibiotc and the emergence of a viable population for sub lethal doses. The elimination of fast growing cells by the antibiotic creates a conducive environment for slow growing cells and their daughter cells (some of which will revert to the fast growing phenotype) due to reduced competition for nutrients.

Our experiments did not constrain nutrients and was rich in both carbon and amino acids. Under these conditions, growth rates and metabolic activity are strongly coupled [32] and is observed in our bioluminescence assay results. Fig 3d–3f show that bioluminescence is strongly and positively correlated with the optical density based estimate of the population for four key combined lactoferrin/*β*-lactam treatments. Firstly, this confirms that the observed biomass is representative of living cells and not a measurement of lysed cells and protein biomass. Since persisters are metabolically frugal, bioluminescence concomitant with observed biomass indicates the presence of metabolically active fast growing cells [33].

Consider the mixed and iron-saturated lactoferrin cases (Fig 3b and 3c), the increase in bacterial growth can be attributed to the loss of lactoferrin’s antibacterial activity. Binding of iron to lactoferrin significantly alters the structural configuration of lactoferrin and leads to the loss of lactoferrin’s antibacterial activities [34, 35]. In addition, *S. aureus* produces siderophores (small molecule iron chelators), and surface proteins like IsdA and a variety of proteases under sub-inhibitory concentrations of antibiotics [36, 37]. Siderophores acquire iron from the extracellular space, and from Fe^3+^ saturated lactoferrin to help the bacteria grow [37]. Surface proteins like IsdA could further reduce the bactericidal activity of lactoferrin [38] resulting in reduced antibiotic efficacy and a larger resilient bacterial population. These results suggest that iron restriction is an essential mechanism through which lactoferrin induces bacteriostasis. Although, iron-bound lactoferrin improves the antibiotic’s efficacy, an increased amount of antibiotic is required. Results from our mathematical modelling, introduced momentarily, suggests that iron-bound lactoferrin continues to play a role in reducing *β*-lactamase production and hence improve efficacy.

These results imply that, in the absence of an antibiotic the bacterial population grows to the carrying capacity of the medium. When treated with a *β*-lactam antibiotic they exhibit adaptive response i.e. bacteria degrade the antibiotic through their secreted *β*-lactamase [39, 40]. At higher concentrations of the antibiotic the bacteria are unable to degrade all the bound antibiotic and eventually lyse. Studies have shown that at intermediate antibiotic concentrations lysed bacteria will release further *β*-lactamase into extracellular space, which confers resilience to the wider population by degrading more antibiotics and signalling the population to release more *β*-lactamase into the extracellular space [41–44]. In the following section we show that these observations are substantiated by our mathematical model.

In summary our experimental results suggest that 1) *S. aureus* cultures display both adaptive and phenotype switching response as observed in biofilms, and 2) this response is dose dependent with sub-optimal doses of lactoferrin leading to increased growth.

### Temporal dynamics of *S. aureus* colonies in response to Cefazolin and lactoferrin treatment

To investigate the role played by the adaptive response and phenotype switching in conferring tolerance to the bacterial population, we used the kinetic model (Fig 1) to infer the temporal population dynamics and the impact of lactoferrin/ *β*-lactam on the bacterial population. Simulations were initialised with a known initial density of susceptible (0.3) and persisters (0.01) and simulated until the population density was above 50% of the carrying capacity (*B*_50_) of the medium. The population was then subjected to the treatment. To eliminate any impact of the initial population density on the results, we performed 10000 independent simulations with different bacterial population densities at the time of the treatment. Bacterial population stratification and growth dynamics was determined from the averaged kinetics.

**Fig 4:**
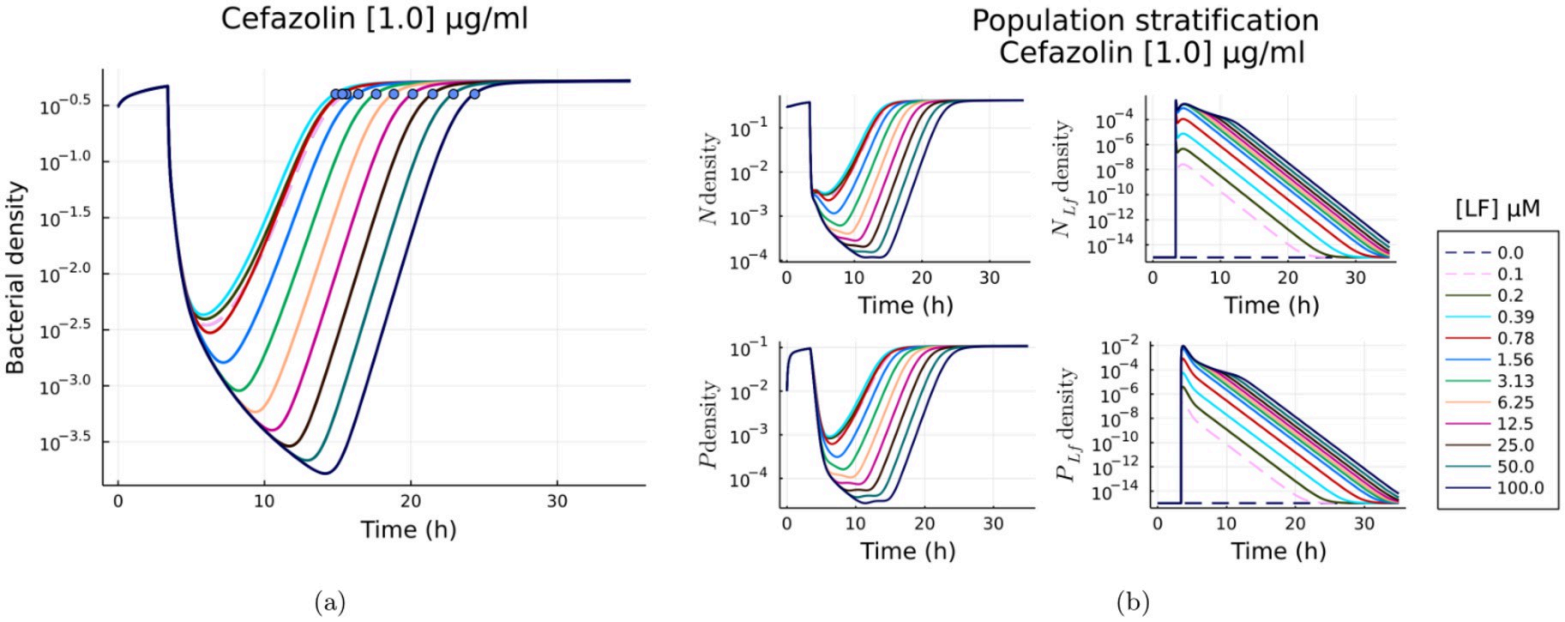
Model predicted population recovery profiles as a function of lactoferrin dose for 1.0 *μ*g/ml Cefazolin. (a) Log density of bacteria, the dots show the times at which the population reaches 50% of the carrying capacity. (b) Log densities of each subpopulation N (susceptible), *N*_*Lf*_ (LF bound susceptible), P (Persister), *P*_*Lf*_ (LF bound Persister).

To quantify the tolerance of the bacteria to recover from lactoferrin/ *β*-lactam dose, we defined, following [24], resilience as the rate of recovery by the population after experiencing the initial crash. An effective lactoferrin/ *β*-lactam treatment will lyse more bacterial cells and occupy the remaining bacteria in degrading the antibiotic. This will result in a prolonged recovery time, and a less effective dose will result in faster recovery, Fig 4a. Based on this, resilience can then be defined as

$$\begin{array}{c} Resilience_{A}=\frac{T_{50}}{T_{50}^{A}} \end{array}$$ (1)

Here *T*_50_, is the time at which the untreated population reaches 50% of its carrying capacity *B*_50_. $T_{50}^{A}$ is the time at which the treated population reaches *B*_50_ post the population crash from antibiotic treatment. The metric will be larger for a resilient response and lower for a non-resilient population.

### Lactoferrin modulates adaptive response

Disruption of *β*-lactamase production is a mechanism that can improve the efficacy of *β*-lactams and can be measured using resilience i.e. low resilience for better efficacy and vice versa. Model results, Fig 4b, show the stratification of bacterial population into lactoferrin bound and lactoferrin free populations of susceptible and persisters in response to treatment. The density of lactoferrin bound bacteria increases proportionally to the lactoferrin dose and affects the time taken by the bacterial population to recover.

**Fig 5:**
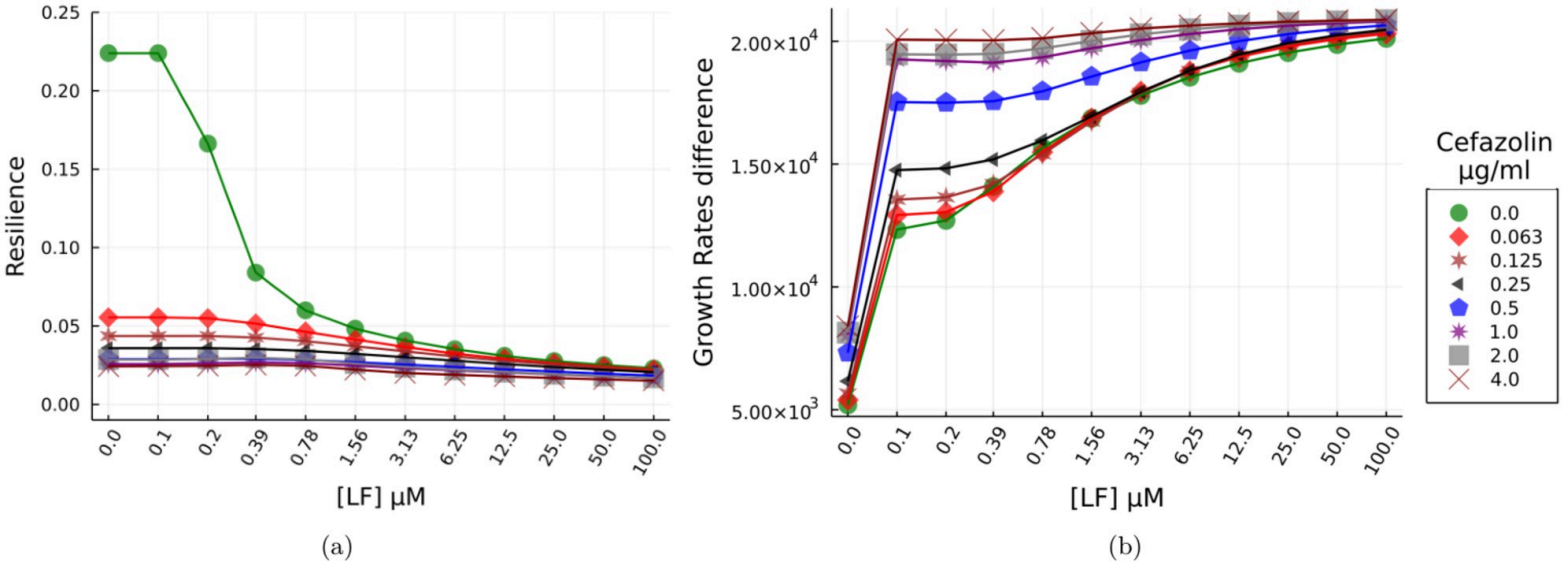
Resilience and differential growth rates of the bacterial population as a function of native lactoferrin/ *β*-lactam concentrations. a) Average resilience data from a stochastic (*N* = 10000) simulation for 8 different doses of Cefazolin, 12 different doses of native lactoferrin, and with different initial susceptible and persister cell fractions are plotted. The variability in the initial population leads to non-unitary value for no treatment condition. b) Difference in maximum growth rates between susceptible (fast growing) and persister (slow growing) cells as function of native lactoferrin/ *β*-lactam concentrations. Values are the average difference between the maximum growth rates for these populations from a stochastic (*N* = 10000) simulation with different initial susceptible and persister cell fractions.

Population resilience was assessed through multiple simulations (*N* = 10000) each with different initial density fractions of susceptible and persister cells at the time of lactoferrin/ *β*-lactam treatment. The population’s growth response for various lactoferrin and *β*-lactam concentrations was predicted and resilience (Eq 1) was computed from the growth kinetics. The averaged resilience for each lactoferrin/*β*-lactam combination is plotted in Fig 5a. The results highlight the key role played by lactoferrin in the bacterial response; while low doses of lactoferrin alone do not significantly degrade the population, larger doses (≥ 1.56 *μ*M) degrade the population to that of minimal sub-inhibitory Cefazolin dose.

### Lactoferrin leads to the selection of fast-growing cells

To investigate the nonlinear effects of lactoferrin dose on the growth rates and bacterial population stratification. We determined from the kinetic model the maximum growth rate of susceptible and persister cells post treatment and computed the difference between these growth rates for different combinations of lactoferrin/ *β*-lactam doses, Fig 5b. This difference characterises the maximum growth divergence that occurs in the population. A large value is indicative of a larger susceptible population and hence more susceptibility to *β*-lactam’s. While a lower value indicates a larger (compared to untreated cohort) persister type cells and greater tolerance and resilience.

In order to eliminate any bias due to the initial population density-fractions of these cell populations. We performed a stochastic (*N* = 10000) simulation with different bacterial phenotype density-fractions for each of the lactoferrin/ *β*-lactam dose at the time of treatment and plotted the average of the difference in susceptible vs persister maximum growth rates. The simulations results show significant growth rate differences being effected in the bacterial sub-populations as a function of lactoferrin dosage. Similar results have been previously reported in [33]; but here as a function of lactoferrin/ *β*-lactam dosage.

Note that, our model assumes that native lactoferrin bound bacterial cells do not divide as lactoferrin inhibits growth. The difference in growth rates shows that lactoferrin treated populations consistently have a larger proportion of susceptible cells compared to persisters and this proportion increases with lactoferrin dose.

## Discussion

Our ability to understand how bacterial populations respond to *β*-lactams in conjunction with other adjuvants is essential to devise combination therapy that would target different resistance mechanisms of bacteria and manifest in more synergistic efficacy, with reduced potential for the emergence of resistant strains. Our experiments show that sub-optimal concentrations of lactoferrin increases bacterial population heterogeneity, sustain growth and limit the efficacy of *β*-lactams. However, optimal doses of lactoferrin improve the efficacy of *β*-lactams by reducing *β*-lactamase production and selection of fast-growing cells that are more sensitive to killing by *β*-lactams. Despite the complexity and numericity of biological processes involved in the population’s response to a treatment, growth rates and the partitioning of population in response to stress provide a level of abstraction that captures the contributions of multiple cell level interactions. We modelled the experimentally observable population dynamics using these abstractions and reveal a role of lactoferrin dose on the population.

Our experiments suggest that dosing regimens that use lower, yet lethal, concentrations of *β*-lactam can be as effective as higher concentrations of *β*-lactam when used with an appropriate lactoferrin dose. This highlights the need for precise understanding of the various bacterial subpopulations and their growth rates in the design of an appropriate lactoferrin/ *β*-lactam dose. Further work needs to be done to resolve the mechanisms by which lactoferrin affects *β*-lactamase expression, whether the bound lactoferrin or its peptides are released in an active form when the bound cells lyse and whether the subpopulations interact differentially with lactoferrin.

Recent insights into human microbiome and its role in general well-being makes it essential that the amount of antibiotic the host is exposed is targeted, minimal and most importantly reduces the emergence of more resistant subpopulation of bacterial pathogens [45, 46]. Our kinetic model uses the bacterial colony’s population level response to a combination antibiotic treatment and predicts recovery times, and effective doses combinations. This information can be used to design dosing regimens based on in-vitro data. It could help in developing effective strategies for both treatment, effective use of *β*-lactam antibiotics and reducing the emergence of antibiotic resistant pathogens.

## Supporting information

S1 Data (XLSX)
