# Correction: Optimal dose of lactoferrin reduces the resilience of in vitro *Staphylococcus aureus* colonies

**DOI:** 10.1371/journal.pone.0307969

**Published:** 2024-08-22

**Authors:** Jagir R. Hussan, Stuart Irwin, Brya G. Matthews, Simon Swift, Dustin L. Williams, Jillian Cornish

The second and third authors name are written incorrectly. The correct names are: Stuart Irwin, Brya G. Matthews. The correct citation is: Hussan JR, Irwin S, Matthews BG, Swift S, Williams DL, Cornish J (2022) Optimal dose of lactoferrin reduces the resilience of in vitro *Staphylococcus aureus* colonies. PLOS ONE 17(8): e0273088. https://doi.org/10.1371/journal.pone.0273088.
